# Exhaled nitric oxide in stable chronic obstructive pulmonary disease

**DOI:** 10.4103/1817-1737.44649

**Published:** 2009

**Authors:** Mohammed F. S. Beg, Mohammad A. Alzoghaibi, Abdullah A. Abba, Syed S. Habib

**Affiliations:** *Department of Physiology, King Saud University and King Khalid University Hospital, Riyadh, Saudi Arabia*; 1 *Department of Medicine, King Saud University and King Khalid University Hospital, Riyadh, Saudi Arabia*

**Keywords:** Asthma, chronic obstructive pulmonary disease, exhaled nitric oxide, stable

## Abstract

**STUDY OBJECTIVE:**

The objective of the study was to test the hypothesis that fraction of exhaled nitric oxide (FENO) is elevated in nonsmoking subjects with stable chronic obstructive pulmonary disease (COPD) and compare it with the results in patients with asthma and a control population.

**DESIGN:**

Cross-sectional study.

**MATERIALS AND METHODS:**

Pulmonology Clinic at a University Hospital. Twenty five control subjects, 25 steroid naïve asthmatics and 14 COPD patients were studied. All the patients were nonsmokers and stable at the time of the study. All subjects completed a questionnaire and underwent spirometry. Exhaled nitric oxide was measured online by chemiluminescence, using single-breath technique.

**RESULTS:**

All the study subjects were males. Subjects with stable COPD had significantly higher values of FENO than controls (56.54±28.01 vs 22.00±6.69; *P*=0.0001) but lower than the subjects with asthma (56.54±28.01 vs 84.78±39.32 *P*=0.0285).The FENO values in COPD subjects were inversely related to the FEV_1_/FVC ratio. There was a significant overlap between the FENO values in COPD and the control subjects.

**CONCLUSION:**

There is a significant elevation in FENO in patients with stable COPD, but the elevation is less than in asthmatic subjects. Its value in clinical practice may be limited by the significant overlap with control subjects.

## Introduction

Nitric oxide (NO) is a molecular gas, which, in the respiratory system, regulates vascular and bronchial tone leading to dilatation, facilitates the beating of the cilia and acts as a neurotransmitter.[1–3] Although NO is formed by different mechanisms and by various types of cells, the larger central airways is believed to be the main source of exhaled NO (eNO) in humans.[4,5] It was first demonstrated in 1991 that NO could be detected in the exhaled gas of animals and humans in the range of three to 20 parts per billion (ppb), by using chemiluminescence analysis.[6] The measurement of fraction of exhaled nitric oxide (FENO) is, however, affected by a number of factors and varies in disease and health. Although the measurement simple, noninvasive and highly reproducible, it is necessary to pay special attention to the technique. The recommendations by the American Thoracic Society (ATS) and the European Respiratory Society (ERS) have standardized the procedure for the offline and online measurements of eNO,[7] thus making the figures comparable between different research centers. Furthermore, the approval of a portable FENO monitoring device by the United States Food and Drug Administration in 2003 may increase the clinical availability and use of this technology.[8] The measurement of exhaled nitric oxide may, therefore, be an integral component of the care of patients with various respiratory conditions.

Nitric oxide plays an important role as an inflammatory mediator in the airways. The fraction of eNO has been used in asthma to establish the correct diagnosis in steroid naïve patients,[9,10] predict favorable response to corticosteroids,[11,12] titrate anti-inflammatory medications,[13,14] predict impending asthma exacerbation[15,16] and monitor adherence to medications.[17,18] A recent trial suggested that care guided by FENO measurements may achieve similar asthma control at lower steroid doses, as compared to guideline-based management.[19] Thus FENO, which is easily measured, is fast being included in the diagnosis and management of asthma. On the other hand, there are only a few studies describing the levels of FENO in patients with COPD and some of the published reports are conflicting in their conclusions. Also, while some studies reported an increase in the values in patients with stable COPD, others have shown reduced or unchanged values.[20–27] Only one of the studies included patients with asthma as a comparator group.[21]

Recent studies indicate the following:

- Patients with COPD may respond differently to treatment, depending on the levels of FENO.[28]
- Increased values of FENO may signify exacerbations.[29]
- Levels correlate with disease severity.[23]

Assessing FENO may, therefore, play an important role in the evaluation, management and prediction of outcome in patients with COPD, as it has done in the case of asthma patients.

This study was, therefore, conducted to compare FENO levels between former smokers with stable COPD; nonsmoking, steroid-naive asthmatics; and, healthy nonsmoking volunteers.

## Materials and Methods

This cross sectional observational study was conducted at the departments of Physiology and Medicine, at the King Khalid University Hospital, King Saud University, Riyadh, Saudi Arabia, from September 2006 to August 2007. Written informed consent was obtained from all the patients and the project was approved by the College of Medicine Ethics Review board.

Seventy five individuals were studied. A total of 64 individuals, who fulfilled the selection criteria were selected for the study - 25 patients with bronchial asthma, 14 patients with COPD and 25 normal controls. Patients classified as COPD had airway obstruction with a forced expiratory volume in the first second (FEV_1_) of <80% and FEV_1_/FVC ratio of <70%, an FEV_1_ increase of <200ml and 15% of baseline figures after bronchodilator. In addition, patients should have stopped smoking for at least one year and have had no exacerbation in the two months prior to the study. Patients with known atopy or positive skin tests to common allergens were excluded from this group. Patients with significant sputum and peripheral blood eosinophilia were also excluded. Patients were classified as asthmatics, if they had history of physician-diagnosed asthma, demonstrated airway obstruction with an increase of FEV_1_ of >15% baseline after bronchodilator and had no history of smoking. Measurement of eNO was done at least 12 hours after the use of β2-agonists in patients on this medication. Similarly, all patients with other systemic diseases such as renal, hepatic, thrombotic and collagen vascular diseases were excluded. Chest radiograph was carried out to exclude other respiratory diseases.

The control group included healthy individuals who were non smokers, without any history of thoracic cage or spinal deformities, respiratory diseases or childhood asthma, allergic rhinitis or atopy. They were matched for age, height, weight, body mass index (BMI) and occupation.

The following studies were performed:

### Ventilatory function parameters

Forced expiratory volume in 1 s (FEV_1_), forced vital capacity (FVC), FEV_1_/FVC, peak expiratory flow (PEF), FEF_25_, FEF_50_ and FEF_75_ were measured by the Vitallograph (ALPHA, Ireland). All recordings were made in sitting position. At least three readings were obtained and the best of the three was taken as the final result.

### Exhaled NO measurements

FENO measurements were performed according to the present recommendations of the American Thoracic Society,[7] using a NOX EVA 4000 chemiluminescence analyzer (SERES-FRANCE) with a sensitivity of 1 part per billion (ppb).

Using online visual monitoring, the subjects were asked to inhale from residual volume to total lung capacity (TLC). Then, the subjects performed a slow expiratory vital capacity maneuver, with a constant standardized expiratory flow rate of 0.05 L/sec (± 10%), resulting in an expiration time of about 20 s, into a Teflon cylinder connected to 3-mm Teflon tubing, without clipping the nose.

To exclude nasal NO contamination, a small expiratory resistance of 1 to 2 cm H_2_O was applied. The subjects inspired from atmospheric air and expired in restricted-breath configuration set up.

The expiratory flow rate was measured by a pneumotachograph of data acquisition system BIOPAC MP-100 (Biopac Systems Inc, USA). Plateau levels of FENO against time were determined and expressed as parts per billion (ppb).

Mean exhaled NO concentrations were determined between 5 and 15 s, after the start of the expiration. Three successive recordings at 1 min intervals were made, and the mean was used in the analysis. Nitric oxide concentrations were calibrated two to three times per week, using a standard NO calibration gas.

### Statistical analysis

The data was analyzed using the computer software program, Statistical Package for Social Sciences (SPSS Version11). Data was expressed as mean ± SD for continuous variables and as percentages for categorical variables. The test applied for statistical analysis was Student's t-test. A *P* value of 0.05 was taken as statistically significant and all tests were two tailed. Linear regression analysis was performed between FEV_1_/FVC ratio as the dependant variable, and FENO levels in healthy subjects, asthmatics and COPD patients as independent variable.

## Results

**Figure 1:**
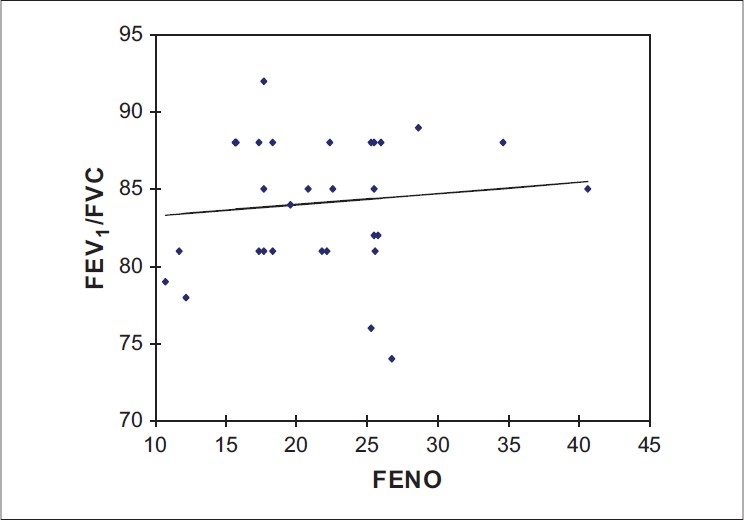
Linear regression analysis showing relationship between FEV_1_/FVC ratio and FENO levels in healthy subjects (r = 0.1079, *P* = 0.5633)

**Figure 2:**
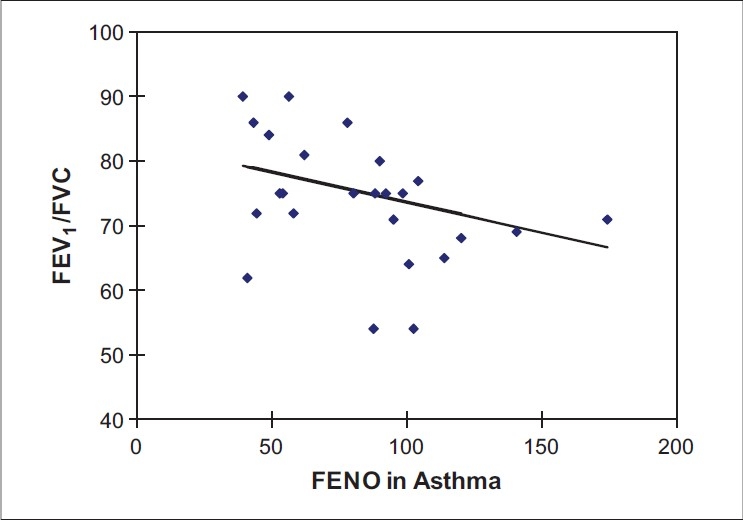
Linear regression analysis showing relationship between FEV_1_/FVC ratio and FENO levels in asthma subjects (r = −0.3076, *P* = 0.1346)

**Figure 3:**
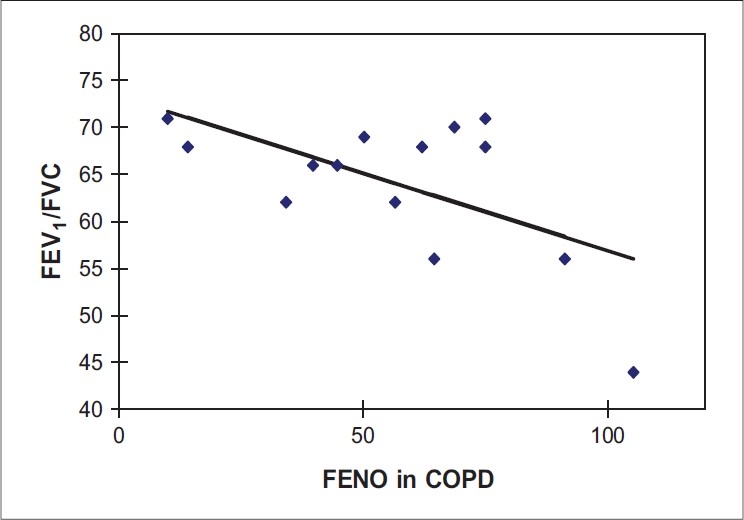
Linear regression analysis showing relationship between FEV_1_/FVC ratio and FENO levels in COPD subjects (r = −0.5855, *P* = 0.0278)

Table 1 gives the clinical characteristics and body composition of control, COPD and asthmatics. The asthmatic patients were younger and had significantly higher body mass index (BMI) than the controls and COPD subjects. The FEV_1_ in liters for healthy, COPD and asthmatic subjects were as follows: 3.64 ± 0.57, 1.75 ± 0.61 and 2.52 ± 0.94 respectively. The FVC in liters for healthy, COPD and asthmatic subjects were: 4.28 ± 0.67, 2.65 ± 0.79, and 3.19 ± 0.96 respectively. The FEV_1_/FVC ratios for healthy, COPD and asthmatic subjects were: 84.75 ± 4.20, 60.69 ± 7.77, and 78.67 ± 10.15 respectively. FENO levels were significantly higher in both asthmatic (84.78 ± 39.32) and COPD (56.54 ± 28.01) subjects, than in healthy subjects (22.0 ± 6.69, *P* = 0.0001). The values of FENO measured in parts per billion (ppb) were higher significantly among the asthmatic subjects, as compared to the COPD subjects, the difference being (84.78 ± 39.32 vs 56.54 ± 28.01 *P* = 0.0285). The results are presented in Figures 1–3 and also Tables 2–4. Furthermore, Figures 1–3 give the linear regression analysis between the FEV_1_/FVC and FENO levels in healthy, asthmatic and COPD subjects.

**Table 1:** Clinical characteristics and body composition of control and COPD patients

	Control *n* = 25	COPD *n* = 14	Asthma *n* = 25
Age (years)	51.78 ± 6.83	54.70 ± 5.87	37.92 ± 14.22
Height (cm)	171.78 ± 4.32	165.20 ± 12.40	175.25 ± 8.14
Weight (kg)	87.73 ± 20.91	91.33 ± 35.50	82.51 ± 15.89
Body mass index	29.54 ± 6.01	26.11 ± 3.44	33.38 ± 7.97*

* *P* < 0.05 vs asthmatics

**Table 2:** Lung function parameters and FENO of control and COPD patients

PFTs	Control *n* = 25	COPD *n* = 14	*P* value
FEV_1_ (Liters)	3.64 ± 0.57	1.75 ± 0.61	0.0000
FVC (Liters)	4.28 ± 0.67	2.65 ± 0.79	0.0001
FEV_1_/FVC	84.75 ± 4.20	60.69 ± 7.77	0.0000
PEF (L/min)	637.50 ± 45.78	249 ± 160	0.0000
FEF25 (L/sec)	8.75 ± 1.33	2.30 ±	0.0000
FEF50 (L/sec)	4.38 ± 0.98	2.44 1.47	0.0281
FEF75 (L/sec)	1.35 ± 0.76	0.40 ± 0.28	0.0000
FENO (ppb)	22.00 ± 6.69	56.54 ± 28.01	0.0000

PFTs-pulmonary function tests; FEV_1_-forced expiratory volume in one second; FVC-forced vital capacity; PEF-peak expiratory flow; FEF25,50,75-forced expiratory flow at 25,50,75% respectively; FENO-fraction of exhaled nitric oxide; ppb-parts per billion

**Table 3:** Lung function parameters and FENO of control and asthma patients

PFTs	Control *n* = 25	Asthma *n* = 25	*P* value
FEV_1_(Liters)	3.64 ± 0.57	2.52 ± 0.94	0.0078
FVC(Liters)	4.28 ± 0.67	3.19 ± 0.96	0.0131
FEV_1_/FVC	84.75 ± 4.20	78.67 ± 10.15	0.1280
PEF(L/min)	637.50 ± 45.78	430.83 ± 213.97	0.0157
FEF25(L/sec)	8.75 ± 1.33	6.28 ± 3.27	0.0674
FEF50(L/sec)	4.38 ± 0.98	3.09 ± 1.56	0.0683
FEF75(L/sec)	1.35 ± 0.76	0.99 ± 0.50	0.2777
FENO(ppb)	22.00 ± 6.69	84.78 ± 39.32	0.0000

PFTs-pulmonary function tests; FEV_1_-forced expiratory volume in one second; FVC-forced vital capacity; PEF-peak expiratory flow; FEF25,50,75-forced expiratory flow at 25,50,75% respectively; FENO-fraction of exhaled nitric oxide; ppb-parts per billion

**Table 4:** Lung function parameters and FENO of asthma and COPD patients

PFTs	COPD *n* = 14	Asthma *n* = 25	*P* value
FEV_1_(Liters)	1.75 ± 0.61	2.52 ± 0.94	0.0230
FVC(Liters)	2.65 ± 0.79	3.19 ± 0.96	0.1345
FEV_1_/FVC	60.69 ± 7.77	78.67 ± 10.15	0.0004
PEF(L/min)	249 ± 160	430.83 ± 213.97	0.0688
FEF25(L/sec)	2.30 ± 0.83	6.28 ± 3.27	0.0014
FEF50(L/sec)	2.44 ± 1.47	3.09 ± 1.56	0.0154
FEF75(L/sec)	0.40 ± 0.28	0.99 ± 0.50	0.0012
FENO(ppb)	56.54 ± 28.01	84.78 ± 39.32	0.0285

PFTs-pulmonary function tests; FEV_1_-forced expiratory volume in one second; FVC-forced vital capacity; PEF-peak expiratory flow; FEF25,50,75-forced expiratory flow at 25,50,75% respectively; FENO-fraction of exhaled nitric oxide; ppb-parts per billion

Among patients with COPD, there was a negative correlation between the FEV_1_/FVC ratio and the level of FENO. The values of FENO among COPD, asthmatic and control ranged between 14-105, 42-175, and 3-40 ppb respectively. While there was no overlap between the FENO figures among asthma and healthy subjects, a number of subjects with COPD had figures within the range found among healthy subjects.

## Discussion

The most significant finding in this study is that FENO is elevated in nonsmoking, nonatopic patients with stable COPD and that this elevation is less than that seen in patients with asthma. Furthermore, there is a significant overlap in the values in subjects with COPD and normal subjects. There is also a correlation between the level of FENO and the FEV_1_/FVC ratio in COPD subjects, but not in asthmatics or normal subjects. Normal FENO levels have not been defined for our population; but, in the selection of controls, all possible confounding factors were taken into consideration. These include smoking habits, height, weight, age and atopy, which are known to be independently associated with FENO.[30,31]

Only steroid naïve patients were included in this study, as use of corticosteroids is known to reduce the levels of FENO.[32–34] The use of β2-agonists is known to increase the levels of FENO.[35] Therefore, all patients included in this study abstained from the use of these agents for at least 12 hours before the measurement of FENO levels. This study is consistent with the findings of others, which showed elevated levels in patients with stable COPD.[21,22] Some studies, however, have yielded conflicting results.[23–25,36]

In COPD, there is accumulation of inflammatory mucous exudates in the lumen and infiltration of the small airway wall by inflammatory cells, as the disease progresses.[37] There is a high level expression of inducible NO synthase (iNOS) present in sputum macrophages alveolar walls, small airway epithelium and vascular smooth muscle of these patients.[38,39] This may result in an increased production of NO and NO-related species in the lung periphery. Although the levels of FENO are elevated in both asthma and COPD, the levels are much higher in the latter group. There is data to suggest that the factors leading to the elevation of FENO in COPD are different from those in asthma.[21] As an example, although there is a relationship between the use of corticosteroids and levels of FENO in asthma,[32–34] no such relationship exists in COPD.[21] There is, however, a relationship between the levels of FENO in COPD and measures of lung function abnormality, as shown by Ansarin *et al*.[21] Exhaled NO inversely correlated with FEV_1_, D_L_ CO and SaO_2_, and was positively correlated with the residual lung volume/total lung capacity ratio in their study. It is also known that the progression of COPD from GOLD stage 0 to 4 is most strongly associated with thickening of the wall of small airways by a repair or remodeling process.[40] Early authors suggest an alternative explanation for the elevation of FENO levels in patients with diffuse pulmonary damage, secondary to years of tobacco smoking. Nitric oxide is a highly reactive chemical, with a short half-life within the body. At the alveolar level, NO combines rapidly with reduced haemoglobin and is, therefore, scavenged by pulmonary capillary blood. In the presence of altered ventilation-perfusion ratio, this scavenging will take place less efficiently, leading to higher levels of exhaled NO. In consonance with our study, Brindicci *et al.*, have shown a significant correlation between alveolar NO (Calv,NO) and both FEV_1_ and FEV_1_/FVC ratio, which was not seen in either healthy subjects or in mild asthmatics.[22] It is suggested that Calv,NO in COPD patients may reflect peripheral inflammation and remodeling, resulting in increased peripheral resistance. This implies that the level of FENO may be an early and simple indicator of the degree of lung damage (ventilation perfusion mismatch) and, therefore, a powerful potential tool in diagnosing early stages of peripheral inflammation in COPD.

Another possible explanation for the elevation in stable COPD patients is the possibility of colonization with micro-organisms which stimulate NO synthase and lead to higher levels of FENO. This is the presumed explanation for the elevation in bronchiectasis.

Our study differs from other studies that noted low or normal levels of FENO. Rutgers *et al*. compared eNO in 16 subjects with COPD to eight healthy non-smokers and found no difference between the two groups.[24] Similarly, Robbins *et al.*, in a comparison of 14 subjects with COPD and 23 healthy controls, found no difference in the eNO levels between the groups.[41] In a study of 17 subjects with exacerbation of COPD, Agusti *et al.* demonstrated an increase in FENO during the period of exacerbation, which remained high even after improvement of symptoms but was comparable to control subjects when patients were in a stable state.[29] One explanation for this disparity with our study may be the differences in the technique of measuring FENO from the lower airways. The level of FENO is affected by prior therapy, flow rate and testing conditions. Because the pathology in COPD is mainly in the small airways and the lung parenchyma, techniques that involve measuring FENO from the larger upper airways may underestimate the levels. Single expiratory technique measures predominantly larger airway derived NO and may only partially reflect peripheral inflammation.[27,42] Brindicci *et al*., by measuring FENO at multiple expired flows, have shown that alveolar FENO is elevated and that it positively correlates with the GOLD Stage. Strict adherence to the recently released American Thoracic Society and European Respiratory Society recommendations for the offline and online measurement of FENO may lead to standardization of methods, and, hence, comparable results.[7] This is the first such comparative study since the recommendations.

A second possible explanation for the differences may be the inclusion of current smokers among patient or control populations. Smoking is well-known to reduce FENO values, probably by down-regulation of endothelial NO synthase (eNOS) and inducible (iNOS).[43,44]

Inclusion of patients who are on steroids and in whom the FENO may be reduced is also another possible explanation. Interestingly, of the 10 subjects followed up by Agusti *et al*. in a stable state,[29] only the three that had FENO levels above the control figures were not on steroids, indicating that the suppression of FENO in the rest was due to the steroids. All our subjects were steroids-naïve.

Another important finding from this study is that there is considerable overlap between the measured values of eNo in normal and COPD subjects. The implication is that FENO values alone may not be useful in differentiating between the two. Rather, establishing different cut points for different clinical settings is more useful and practical. Our study is, however, limited by size and more comparative studies need to be conducted.

In conclusion, this study corroborates other studies that FENO is increased in stable, nonsmoking COPD but that the levels are lower than in asthma. The considerable overlap between the values seen COPD and controls precludes its use as a diagnostic tool. Nevertheless, the development of new hand-held analyzers will make the measurement more readily available, make larger comparative studies possible and hence make possible application in day to day practice.
